# Randomised trials relevant to mental health conducted in low and middle-income countries: a survey

**DOI:** 10.1186/1471-244X-8-69

**Published:** 2008-08-14

**Authors:** Rebecca J Syed Sheriff, Clive E Adams, Prathap Tharyan, Mahesh Jayaram, Lelia Duley, the PRACTIHC Mental Health Group

**Affiliations:** 1 Unidad Epidemiologia Clinica, Hospital San Ignacio, Santafe de Bogota, Colombia; 2 Cochrane Schizophrenia Group, Division of Psychiatry, University of Nottingham, UK; 3 South Asian Cochrane Network; Prof. B V Moses & Indian Council of Medical Research Advanced Centre for Research and Training in Evidence Based Health Care, Christian Medical College, Vellore, India; 4 Leeds Partnerships Foundation Trust, Leeds, UK; 5 Centre for Epidemiology and Biostatistics, University of Leeds, UK; 6 Health Services and Population Research Department, Institute of Psychiatry (Kings College London), De Crespigny Park, Denmark Hill, London, SE5 8AF, UK

## Abstract

**Background:**

A substantial proportion of the psychiatric burden of disease falls on the world's poorest nations, yet relatively little is known about randomised trials conducted in these countries. Our aim was to identify and describe a representative sample of mental health trials from low and middle-income countries.

**Methods:**

6107 electronic records, most with full text copies, were available following extensive searches for randomised or potentially randomised trials from low and middle-income countries published in 1991, 1995 and 2000. These records were searched to identify studies relevant to mental health. Data on study characteristics were extracted from the full text copies.

**Results:**

Trials relevant to mental health were reported in only 3% of the records. 176 records reporting 177 trials were identified: 25 were published in 1991, 45 in 1995, and 106 in 2000. Participants from China were represented in 46% of trials described. 68% of trials had <100 participants. The method of sequence generation was described in less than 20% of reports and adequate concealment of allocation was described in only 12% of reports. Participants were most frequently adults with unipolar depression (36/177) or schizophrenia (36/177). 80% of studies evaluated pharmacological interventions, a third of which were not listed by WHO as essential drugs. 41% of reports were indexed on PubMed; this proportion decreased from 68% in 1991 to 32% in 2000.

**Conclusion:**

In terms of overall health burden, trial research activity from low and middle-income countries in mental health appears to be low, and in no area adequately reflects need.

## Background

Most of the global burden of mental illness falls to the poorest nations, where 80% of world's population live [1]. On average low and middle-income countries devote less than 1% of their health expenditure to mental health and have poorly developed mental health policies and legislation. Treatment provision is often dismally under resourced [2]. Randomised trials are the gold-standard for evaluation of care, and systematic reviews of randomised trials increasingly provide the basis for health care practice and policy. Most trials, however, are conducted in high-income countries [3]. The interventions assessed may be unaffordable, unavailable or inappropriate for people in other cultures and settings [4].

The extent to which interventions relevant to the prevention and treatment of mental health problems in low and middle-income countries have been evaluated locally is unclear.

As part of an earlier project [5] bibliographic databases were extensively searched to identify randomised, and possibly randomised, trials with participants from low or middle-income countries published during 1991, 1995 or 2000. The project presented here aimed to describe the content and quality of mental health trials within this dataset.

## Methods

### Search strategy

Detailed methods for this study have been published previously in this journal [6]. In brief, an earlier project searched the Cochrane Library's Central Register of Controlled Trials (CENTRAL) plus 25 biomedical bibliographic databases to identify randomised or possibly randomised trials from low and middle-income countries published in 1991, 1995 or 2000. Countries were classified using World Bank definitions, categorizing by Gross National Income per capita and by geographical region [7]. For this paper, the electronic records of these initial searches were searched again to identify studies relevant to mental health. These records included titles, keywords, and, where available, abstracts. Word roots based on Mesh headings within "Mental Health" were utilised with extra terms in Spanish, Portuguese and French. In addition, for 10% of the records not identified as being relevant to mental health by the electronic search, full text copies were checked to assess whether this was truly the case. For each study identified as potentially relevant to mental health, the author names were checked in PubMed for additional citations from the three sample years (1991, 1995 and 2000). Full text copies for all trials potentially relevant to mental health were sought.

### Assessment of studies for inclusion

The criteria used to assess eligibility were:

(i) Randomised or possibly randomised trials – this included randomised trials, quasi randomised studies, and controlled trials which could possibly be randomised.

(ii) Published in 1991, 1995 or 2000.

(iii) At least one participant from a low or middle-income country as stated in the text, or if not stated this was assumed if reports were from databases local to low and middle-income countries and reported in local languages.

(iv) Participants were either people who had a mental health problem, or they had been identified as being at increased risk of developing a mental health problem.

Two authors (RJSS and MJ) independently assessed full text copies of each record identified by the search. Discrepancies were resolved by discussion.

### Data extraction and data entry

Data on study quality and content were extracted onto a specifically designed data extraction form. Each trial was extracted onto a separate data extraction sheet. Hence, if two trials were reported in the same paper, these were extracted separately. For English language reports, data were extracted by two people working independently. For studies in other languages data were extracted twice for a random sample of 10% of papers. Extraction was by someone fluent in the relevant language. In the few cases where full text was not available (n = 12), data were extracted from the title plus abstract (n = 10), or title alone if that was all that was available (n = 2).

Health conditions of participants in the trials were categorised using the broad categories of the International Classification of Diseases tenth edition (ICD-10). The interventions being evaluated were classified as drug, physical non-drug, such as Electro-Convulsive Therapy (ECT) or psychosocial. Drugs were further classified as to whether they are listed on the WHO List of Essential Medicines [8]. Outcomes were classified using a system developed for a survey of trials of schizophrenia [9].

Data were entered into Meerkat software [10] and compared. For each variable, 80% agreement was considered acceptable. If the agreement fell below this, possible reasons were investigated and data re-extracted. To assess accessibility, each citation was searched for on PubMed in August 2006 using author AND year or failing this title.

### Statistical analyses

Characteristics of the trials in terms of where they were conducted, the language of report, health problems being addressed, interventions and outcomes were described. Frequencies were compared using χ^2 ^for linear trend. Topics covered by the trials were compared, by region, with the WHO revised 2002 burden of disease estimates [11].

Global burden of disease estimates for mortality and YLD (years lost to disability) are available for both economic groups (high, upper middle, lower middle and low) and broad geographic regions [12]. These estimates are further categorised by cause e.g. unipolar depression. We calculated an estimate of how trial-based research activity reflects burden of disease as a 'research: need' ratio, by dividing the number of people randomised with each category of mental health problem by the WHO's Years Lost due to Disability (YLD) for the same category. The findings were compared with each other in order to investigate which disorders and populations are the focus of a relatively high or low research activity.

## Results

Of the 5838 records not identified as being potentially relevant to mental health by electronic searching, full reports of 587 records in English, 380 in Spanish or Portuguese and 144 in Russian, Ukrainian or Romanian were hand searched to check if this was truly the case. An extra three reports were identified as being potentially relevant to mental health and subject to independent eligibility assessment. Only one of the three studies was deemed eligible for inclusion in this survey.

Of the 6107 reports of randomised or possibly randomised studies with participants in low and middle-income countries, and published in the years 1991, 1995, and 2000, 176 (3%) were relevant to mental health.

Of the 176 records, 167 represented full reports in a journal; for five of these we had access to the abstract only. Two of the full journal reports described multiple trials: for one of these reports only one trial was relevant to mental health, for the other report two trials were relevant. Of the other records; two were conference abstracts, one was a conference proceeding, three were journal letters and one was a thesis. For a further two citations, only the title was available.

As one report described two trials, 177 studies are included in the analyses presented here. Of these, a full text report was available for 165 (93%). Agreement for data extraction exceeded 80% for all items.

The number of reports increased across time with 25 in 1991, 45 in 1995, and 107 in 2000. Overall, 85% of the reports were written in either English (80/177) or Mandarin Chinese (71/177). The proportion of reports written in Chinese increased from a quarter (7/25) to a half (53/107) over the sample period. The languages of the remaining 15% of reports in descending order of frequency were Spanish, Russian, Portuguese, Korean, Polish and Serbo-Croatian. For one citation for which we had only obtained the title the language of the full report remains unknown.

**Figure 1:**
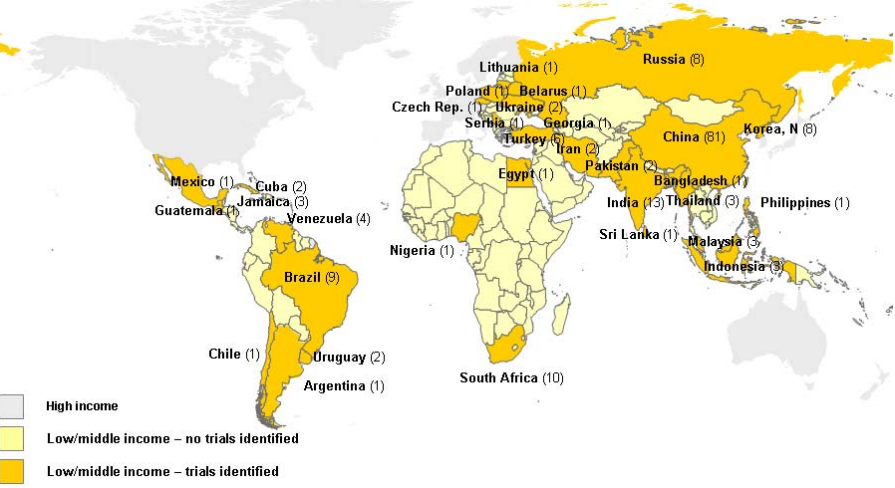
Number of trials by country (totals for sample period).

A quarter of the trials recruited from low-income countries (41/177) (Figure 1). China was the country of recruitment for 46% of the studies (81/177); it was a low-income country in 1991 and 1995, but had become lower middle-income in 2000. Seven trials recruited from more than one country, with two including a high-income nation. Two thirds of the trials (118/177) had less than 100 participants, 30% (52/177) randomised 100–499 people and 3 studies had more than 500 participants. The proportion of studies with less than 100 participants did not change substantively across time. Setting for the study could be determined from the report for 82/177 studies (46%). Of these, 68 were urban, 7 were rural and 7 were both. The site of recruitment was not clear for 41/177 studies (23%). Of the 136 reports with relevant information on site, this was a hospital for 82% (111/136), in the community for 8% (11/136), in an educational institution for 5% (7/136), in primary care for 1% (2/136), and in a variety of other sites for 4% (5/136).

### Study quality and funding

Adequate allocation concealment was described for 12% of studies (21/177), and method of sequence generation was stated for 17% (30/177). For 62% of studies (109/177) there was information about blinding of the intervention; 77/109 were reported to have been double blind. One in five reports mentioned an ethics committee (34/177), and a third mentioned consent (61/177). A source of funding was mentioned for 25% of studies (45/177).

### Participants

People aged 18–60 years were included in 79% of the studies (135/177). Of these, 39 also included participants who were over 60 years, and 12 also included those who were younger than 18 years. Seven studies included only people aged more than 60 years, and 22 included only people younger than 18 years. Age of the participants was not clear in 13 trials (7%). People of both sexes were recruited to three quarters of the trials (135/177). Participants were male only for 19 studies and female only for 7. Gender of participants was not clear for 25 reports (15%).

The most common health problems for participants in the studies were unipolar depression, (36/177) and schizophrenia (36/177) (Table 1). Three trials (1%) addressed prevention rather than treatment.

**Table 1:** Mental health problems assessed, by year of publication

**Mental Health Problem**	**1991**	**1995**	**2000**	**Total**
	**n = 25 (%)**	**n = 45 (%)**	**N = 107 (%)**	**N = 177 (%)**
Unipolar depression	6 (24)	3 (7)	27* (25)	**36 (20)**
Schizophrenia	6 (24)	9 (20)	21 (20)	**36 (20)**
Other anxiety disorders	3 (12)	3 (7)	14 (13)	**19 (11)**
Alzheimer's and other dementias	3 (12)	4 (9)	12* (11)	**19 (11)**
Disorders associated with brain damage/dysfunction	-	9 (20)	5 (5)	**14 (8)**
Drug use disorders	2 (8)	3 (7)	8 (7)	**13 (7)**
Bipolar depression	-	4 (9)	5 (5)	**9 (5)**
Psycho sexual disorders	2 (8)	1 (2)	5 (5)	**8 (5)**
Alcohol use disorders	2 (8)	4 (9)	1 (<1)	**7 (4)**
Panic disorder	-	1 (2)	5 (5)	**6 (3)**
Obsessive Compulsive Disorder	-	1 (2)	2 (2)	**3 (2)**
Non organic enuresis	1 (4)	1 (2)	-	**2 (1)**
Non organic sleep disorders	-	1 (2)	1 (<1)	**2 (1)**
Somatoform disorders		1 (2)		**1 (<1)**
Conduct disorder	-	-	1 (<1)	**1 (<1)**

* includes prevention trials

### Interventions and outcomes

Most studies (80%) evaluated pharmacological interventions (Table 2); a third of these drugs (47/140) were listed by WHO as essential drugs.

**Table 2:** Types of intervention trial and year of publication

**Type of trial**	**1991**	**1995**	**2000**	**Total**
	**n = 25 (%)**	**n = 45 (%)**	**n = 107 (%)**	**n = 177 (%)**
Drug vs drug	*13 (52)*	*20 (44)*	*56 (52)*	***89 (50)***
Drug vs non-active (eg placebo)	4 (16)	9 (20)	20 (19)	**33 (19)**
Drug vs psychosocial	-	-	7 (7)	**7 (4)**
Drug vs biological non-drug (eg ECT)	2 (8)	2 (4)	2 (2)	**6 (3)**
Drug vs mixed (eg placebo AND psychotherapy)	-	-	1 (<1)	**1 (<1)**
Drug dose finding	1 (4)	2 (4)	1 (<1)	**4 (2)**
**Total trials involving drugs**	**20 (80)**	**33 (73)**	**87 (81)**	**140 (80)**
Biological non-drug vs biological non-drug	1 (4)	2 (4)	3 (3)	**6 (3)**
Psychosocial vs psychosocial	*4 (16)*	*4 (9)*	*5 (5)*	***13 (7)***
Psychosocial vs non-active		3 (7)	8 (7)	**11 (6)**
Biological non-drug vs non-active		1 (2)	3 (5)	**4 (2)**

ECT = electroconvulsive therapy

Mental state was the most common outcome, reported by three quarters of studies (129/177) (Table 3). Less than half the studies reported adverse effects (78/177). For 83% of studies (147/177) at least one scale was used to rate outcome (median = 1, range = 1–6). The most frequently reported scales were Hamilton rating scale for depression (HAM-D) (24/177), and the Hamilton Anxiety Scale (HAM-A) (18/177). Nine reports did not include data on outcome.

**Table 3:** Types of outcome reported, by year of publication

**Outcome**	**1991**	**1995**	**2000**	**Total (%)**	**Number of different scales**
	**n = 25**	**n = 45**	**n = 107**	**n = 177**	
Mental state	12	26	91	**129 (73)**	**26**
Physiological assessment	5	24	52	**81 (46)**	**15**
Adverse effects	4	19	55	**78 (44)**	**5**
Global impression	5	9	28	**42 (24)**	**6**
Cognitive functioning	6	11	21	**38 (21)**	**7**
Withdrawal and craving	-	3	9	**12 (7)**	**3**
Psychological tests	1	6	2	**9 (5)**	**8**
Social functioning	-	3	5	**8 (5)**	**4**
Behaviour	-	1	7	**8 (5)**	**1**
Development and learning		3	3	**6 (4)**	**1**
Compliance/attitudes to treatment	1	-	3	**4 (2)**	**2**
Quality of life	-	-	2	**2 (1)**	**1**
Economic	1	-	-	**1 (1)**	**-**
Use of health service resources	-	-	-	**-**	**-**

Studies reported more than one type of outcome

### Accessibility

Overall, 41% of the reports could be identified on PubMed when searched in August 2006 (73/177). The proportion identified decreased from 68% in 1991 to 34% in 2000 (χ^2 ^for linear trend 10.92, 1 df, p = 0.001). Of the 80 reports in English, 54 (68%) were identified in PubMed, compared with nine (13%) of the 71 reports in Chinese. The proportion of Chinese reports identified on PubMed decreased from (6/7) 86% in 1991 to (2/53) 3% in 2000. In total 10 (40%) of the 25 reports in other languages were identified in MEDLINE. 66% (117/177) of citations were identified on Cochrane's CENTRAL during the original search, again the proportion located dropped from 80% in 1991 to 57% in 2000.

### Ratio of trial-based research activity to burden of disease

Unipolar depressive disorders and schizophrenia have the highest burden of disease as assessed by years lost due to disability (Table 4). However, more people with drug use disorders were randomised per year lost due to disability than any other type of mental health disorder.

**Table 4:** Research:need ratios for all sample years

**Mental health problem**	**Number of trials**	**Number of participants**	**WHO YLD 2002**	**Ratio**
Unipolar depressive disorders	36	2913	56484904	**5.16**
Schizophrenia	33	5270	14353406	**36.72**
Alcohol use disorders	7	434	13646427	**3.18**
Bipolar disorder	9	805	12472021	**6.45**
Panic disorder	6	272	5996378	**4.54**
Dementias	19	1418	5421155	**26.20**
Obsessive-compulsive disorder	3	202	4364394	**4.63**
Drug use disorders	13	5749	4064053	**141.40**
Post traumatic stress disorder	0	0	2842102	**-----**

Mental Health Problems were sorted by WHO YLD 2002

Ratio = Number randomised/YLD need (×10*6)

## Discussion

Over the decade included in our sample, only 3% of trials from low and middle-income countries focused on mental health problems. The quality of reporting of study design was poor, with the procedures used for randomisation and concealment of allocation being described for less than one fifth. Inadequate reporting of the methodological quality of trials relevant to mental health is, however, also common for studies conducted in Europe and in North America [9]. Even in high impact general journals, 40% of studies fail to describe the procedures used for randomisation [13]. Two thirds of the studies in our sample mentioned blinding of the intervention, although we did not attempt to assess the adequacy or completeness of this information. It seems unlikely that many were adequately blinded, as, in trials for people with schizophrenia, just over one fifth have adequate descriptions [9].

### Size

Most trials were small and few involved more than one country. The value of international trials has been demonstrated in cancer, heart disease and perinatal care; such studies enable large numbers to be recruited [14], ensure wide generalisability of results [15] and facilitate research capacity building [16]. Conducting large international trials in mental health would be likely to have similar advantages.

### Content

Participants in the trials reported here had a wide range of mental health problems, but the most common were depression and schizophrenia. Overall, 80% of the studies evaluated the effects of one or more drugs. This is the same level as for trials of schizophrenia overall [9], and hence may reflect a greater acceptance of quantitative evaluation for drugs as compared to other forms of therapy within mental health. The low level of community based and prevention studies highlights a major gap for future research. Such trials should be a priority.

### Outcomes

Most studies reported using scales to assess outcomes. Nevertheless, interpretation of scales can be difficult and they are rarely used in clinical care. It would therefore be more relevant for trials to report outcome measures that have more intuitive meaning to patients, their families, and clinicians. It would seem a shame for those rare trialists in low and middle-income countries to dissipate their energies recording outcomes that are difficult to use in everyday practice.

### Reporting

The low level of reporting on how the study was funded is not uncommon. For example, funding source was reported for 39% to 51% of surgical trials [17,18]. Similarly, many studies describing the quality of randomised trials do not report on whether ethics approval and consent are explicitly mentioned.

The CONSORT (Consolidated Standards of Reporting Trials) statement of 1996 [19], updated in 2001 [20] gives recommendations for how to report randomised trials using a 22 item checklist. Some journals have adopted CONSORT; although this endorsement is associated with improved reporting of randomised trials, reporting is often still not adequate [13,21]. Considerable scope remains for improving the reporting of randomised trials. In 2003, only 22% of high impact general and internal medicine journals mentioned CONSORT in their instructions for authors, and many used ambiguous language regarding what was expected from authors [22]. Nevertheless, as funding source, ethics approval and consent are not included in the CONSORT checklist, the quality of reporting for these items may not improve unless researchers are made more aware of their relevance.

### Geographical Variation

The proportion of trials from different geographic regions varied greatly; for example only three trials were identified from the Middle East or North Africa region (comprised of Algeria, Djibouti, Egypt, Iran, Iraq, Jordan, Lebanon, Libyan Arab Jamahiriya, Malta, Morocco, Occupied Palestinian Territory, Oman, Saudi Arabia, Syrian Arab Republic, Tunisia and Yemen). In this largely Arabic speaking part of the world, with a population of 300 million people, mental health literature may be not be easily accessible by searching in English, and many journals are not indexed on any database [23]. Nevertheless, despite searching African, Arab, Iranian and several Egyptian and Eastern Mediterranean biomedical databases, only three studies were identified. The religious and cultural individuality of this region, along with the presence of conflict, could make evaluative mental health research enormously difficult.

In contrast, China produced almost half (46%) of all trials in mental health, and accounted for most of the increase in annual output across the decade 1991 to 2000. Although the number of mental health trials does seem to be linked to national wealth [24], the increased trial activity in China is unlikely to be driven by income alone. With rapidly increasing numbers of trials there has been concern about the quality of randomised trial conducted in China [25]. Evidence of similar trends [26] are beginning to happen in randomised trials of traditional Chinese medicine which have increased in both quality and quality since 2000 [27].

Since 2001 it appears that China continues to be dominant in its production of mental health trials as evidenced in a recent review of mental health trials from low and middle-income countries where two thirds of trials conducted in low and middle-income countries were from China [28]. This review differs from our survey in that it covers a different time period and has a narrower range of interest concentrating on interventions for schizophrenia, depression, developmental disability and alcohol dependence.

### Accessibility

Forty five percent of these reports were in English. 'Positive' trial reports are more likely to be published in English than in other languages [29]. Less than half of these studies from low and middle-income countries were cited on PubMed; even for reports in English one third were not available in PubMed. Research from low and middle-income countries is therefore underrepresented [30,31]. Proportions of these trials accessible through Cochrane's CENTRAL register are somewhat better. Although CENTRAL is the most comprehensive database of randomised trials, one third of these mental health studies were not available on it.

Although the number of trials conducted in each year is increasing across time, accessibility of their citations on bibliographic databases, such as PubMed, seems to be decreasing. The time difference from publication to our assessment of accessibility was greatest for studies from 1991, nevertheless this seems unlikely to explain the decline in accessibility within PubMed over time, as by 2006 studies published in 2000 should have been indexed and available. The lack of availability on MEDLINE of trials reported in Chinese along with the large increase in trials originating in China seems an important factor. Indexing has been found to be poor with only 95 of the total 4 959 journals which are listed in MEDLINE being published in China [32]. Reports of randomised trials are not always easy to identify, and efforts by institutions such as the Cochrane Collaboration and WHO to make them more accessible should continue.

### Comparison with burden of disease

Using the crude estimates within our sample, more people with 'drug use disorders' (other than alcohol misuse) have been randomised per year-lost-though-disability in low and middle-income countries than people in the other categories of mental health problems. This may reflect the greater availability of funding for research relevant to hepatitis or HIV/AIDS, compared with other mental health problems such as schizophrenia, dementia, affective or anxiety-linked problems, alcohol related difficulties and psychological trauma.

### Suggestions to improve relevance, standards and reporting of trials from low and middle-income countries

Accessibility to trials conducted in low and middle-income countries should be improved by prospective registration of all future trials, as endorsed by the International Committee of Medical Journal Editors (ICMJE) and the World Health Organization's International Clinical Trials Registry Platform (WHO-ICTRP) [33,34]. China, India and Sri Lanka now have national trial registries that form part of the WHO-ICTRP network of Primary Registers [34]. Registers in Latin America and Africa are also preparing to meet international requirements for trial registration [35,36]. Prospective registration as a pre-requisite to publication is also beginning to receive local support from medical journal editors [37].

In addition to improving the accessibility of trial reports prospective registration of trials could also improve the internal validity of trials. By encouraging reporting of elements identified in the CONSORT statement in trial protocols (method of randomization, allocation concealment, blinding and attrition) trial design will be optimised during the planning of trials [38]. Trial registration is also being used in some countries to improve the planning and reporting of ethical considerations such as informed consent, funding sources and ethics committee approval [39].

We would also suggest that coordinated action is required from medical journal editors in low and middle-income countries to adhere to the ICMJE uniform requirements for submitting final reports for trials [40]. This would require details of ethics committees, informed consent and funding sources in addition to the use of CONSORT checklists and flow diagrams. Provision of clear instructions to authors regarding CONSORT and ICMJE requirements and appropriate checklists, education of peer reviewers, and journal policies that consistently preclude publication of trials that do not meet these requirements are also suggested [41].

Initiatives to provide training for ethics committees, researchers, investigators, regulators and students have commenced in some parts of the developing world as a crucial first step in building capacity for ethically sound research [42,43]. However, local and regional regulatory frameworks and legislation are also needed to interpret international guidelines on the standards of care in the context of local constraints.

### Strengths

Strengths of our study are that we utilised comprehensive and extensive electronic searches of not only the Cochrane Library's CENTRAL, but also of biomedical bibliographic databases from low and middle-income countries. Full text copies were available for a high proportion of citations. Previous surveys of mental health research from low and middle-income countries have either concentrated on particular regions or have investigated only selected journals or individual databases [30,31].

### Weaknesses

Despite the extensive searches, it is likely that we failed to identify some relevant studies. Due to time constraints, we were not able to search all the bibliographic databases which include journals from low and middle-income countries, and new databases have become available since our searches were completed. Other factors are that poor indexing within some databases may have impaired retrieval, and some journals may not be indexed or only partially indexed.

## Conclusion

What this paper adds

• The enormous disparity between mental health evaluative research and other areas of medicine

• The emergence of China as a force in this area

• Pioneers are increasingly undertaking research in situations of great constraint

• The increasing difficulty in identifying this work

In terms of overall health burden, trial research activity from low and middle-income countries in mental health appears to be low, and in no area adequately reflects need. Pragmatic randomized trials in mental health addressing locally relevant issues, conducted during routine clinical practice, free of industry sponsorship and using simple, clinically relevant outcomes are emerging from low and middle-income countries [44,45]. However, the results of this survey suggest that studies of population-based, non-drug interventions and those evaluating prevention and effective service delivery are needed. Research conducted in low and middle-income countries should be accessible and address questions of relevance to local health needs and use outcomes that are useful to patients, carers and clinicians based in those countries.

## Competing interests

Lelia Duley is a partner in the Practihc project. Clive Adams, Prathap Tharyan and Lelia Duley have all contributed to the main Practihc project of trials in low and middle-income countries.

## Authors' contributions

RJSS coordinated this study, determined eligibility, extracted data, inputted data, and analysed data and drafted the final report. LD helped design the study and supervised its conduct, helped with reliability checks, manage data and draft the final report. CEA helped design the study, supervised its conduct, undertook searches and managed the resulting datasets, co-ordinated hard copy acquisition and distribution, helped co-ordinate reliability checks, manage data and draft the final report. PT helped design the study and supervised its conduct, helped with reliability checks, manage data and draft the final report. MJ helped determine eligibility and extract data.

## Pre-publication history

The pre-publication history for this paper can be accessed here:


